# Quantitative analysis of cell-type specific gene expression in the green alga *Volvox carteri*

**DOI:** 10.1186/1471-2164-7-321

**Published:** 2006-12-21

**Authors:** Ghazaleh Nematollahi, Arash Kianianmomeni, Armin Hallmann

**Affiliations:** 1 Department of Cellular and Developmental Biology of Plants, University of Bielefeld, Universitätsstr. 25, D-33615 Bielefeld, Germany

## Abstract

**Background:**

The multicellular alga *Volvox carteri *possesses only two cell types: mortal, motile somatic cells and potentially immortal, immotile reproductive cells. It is therefore an attractive model system for studying how cell-autonomous cytodifferentiation is programmed within a genome. Moreover, there are ongoing genome projects both in *Volvox carteri *and in the closely related unicellular alga *Chlamydomonas reinhardtii*. However, gene sequencing is only the beginning. To identify cell-type specific expression and to determine relative expression rates, we evaluate the potential of real-time RT-PCR for quantifying gene transcript levels.

**Results:**

Here we analyze a diversified pool of 39 target genes by real-time RT-PCR for each cell type. This gene pool contains previously known genes with unknown localization of cellular expression, 28 novel genes which are described in this study for the first time, and a few known, cell-type specific genes as a control. The respective gene products are, for instance, part of photosynthesis, cellular regulation, stress response, or transport processes. We provide expression data for all these genes.

**Conclusion:**

The results show that quantitative real-time RT-PCR is a favorable approach to analyze cell-type specific gene expression in *Volvox*, which can be extended to a much larger number of genes or to developmental or metabolic mutants. Our expression data also provide a basis for a detailed analysis of individual, previously unknown, cell-type specifically expressed genes.

## Background

**Figure 1:**
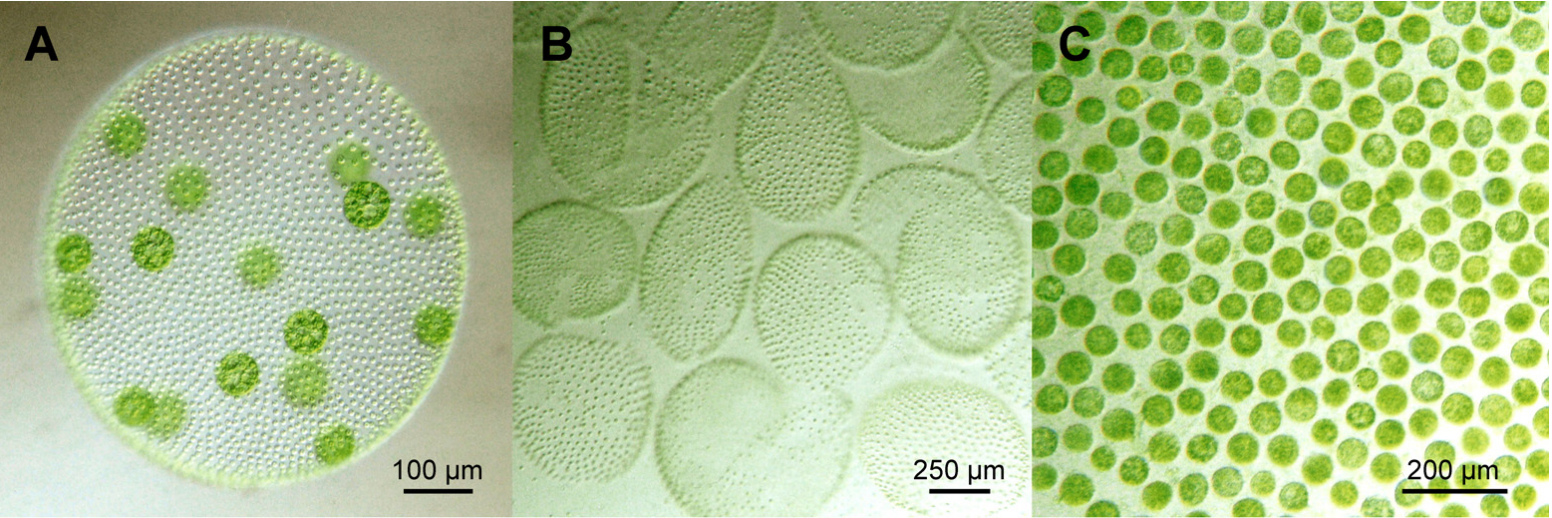
**Phenotype of *Volvox carteri *and appearance of separated cell types**. A) Wild-type phenotype of an asexual female of *Volvox carteri *f. *nagariensis *containing ~2000 small, terminally differentiated somatic cells at the surface and ~16 large reproductive cells (gonidia) in the interior. More than 95% of the volume of such a spheroid consists of a complex but transparent extracellular matrix. B) Isolated somatic cell sheets of *V. carteri*. C) Isolated gonidia of *V. carteri*.

The green alga *Volvox carteri *has a level of complexity representing an ideal model system for studies of multicellularity and cellular differentiation [1,2]; each wild-type *Volvox *spheroid contains only two cell types, somatic cells and reproductive cells (gonidia) (Fig. 1A). Both cell types arise through a sequence of rapid symmetric and asymmetric cleavage divisions of a single gonidium. The two cell types are arranged in a simple, well-defined pattern and are different from each other with respect to physiology, developmental potential, morphology, and size [3]. Not only is the simplicity of *Volvox *auspicious for developmental biologists, but its phylogenetic relationships are also promising: *Volvox *and its simpler, but closely related, unicellular and colonial relatives, the volvocine algae *Chlamydomonas*, *Gonium*, *Pandorina*, *Eudorina *and *Pleodorina*, provide a coherent family of organisms for studying the molecular evolution of multicellularity and cellular differentiation [4]. Another outstanding advantage of volvocine algae is that there are ongoing genome projects both for the multicellular alga *Volvox carteri *and for the unicellular alga *Chlamydomonas reinhardtii*: Shotgun sequencing of both nuclear genomes was performed in each case at approximate 8× coverage by the Joint Genome Institute (JGI, Walnut Creek, CA). For *Chlamydomonas*, extensive cDNA and genomic sequence information has already become publicly available [5], with approximately 90% of the ~120 Mb nuclear genome sequenced; genomic data and data from ~300 k ESTs have been assembled into over 12,000 'unique' cDNAs, and annotation proceeds. Regarding the *Volvox *genome, which is about the same size as the *Chlamydomonas *genome, only shotgun sequences with 1× coverage are publicly available at the moment on the JGI sites, but the completed 8× coverage genomic data will be released before long; also ~80 k ESTs have already been sequenced at JGI and will be released shortly.

Although determination of the sequence of every gene in *Volvox *or any other species allows a better understanding of the organism's physiological potential, it is just the first step of a complete description of how the organism works. One of the next steps should be the determination of mRNA expression levels. Because it is known from many species that much of the transcriptome is compartmentalized and *Volvox *is particularly suitable for studies of multicellularity and cellular differentiation, it is logical to start with an analysis of cell-type specific gene expression, i.e. somatic cells versus gonidia, in order to provide a basis for disclosing cell-specific functions.

In earlier studies, 19 gonidia-specific and 12 somatic-cell-specific cDNAs have already been identified in wild-type *Volvox *by a differential screen of cDNA libraries, and abundance of the transcripts has been analyzed in each of the cell types by Northern blots using radiolabeled restriction-digested DNA as probes [6]; two of these cDNAs/genes have been added to our study as a reference (*gon30*, *gon167*). Furthermore, a couple of interesting developmentally-controlled or cell-type specific genes and their gene products have been identified by generating and analyzing mutants or by Mendelian analysis, e.g. the *lag *gene product (late gonidia), which acts in large pregonidial cells to repress somatic development [4,7,8], and the *regA *gene product (somatic regenerator), which acts on somatic cells to suppress gonidial development [9]. The latter gene was also used as a control gene in our study. Previously, it has also been shown that somatic cells and gonidia display substantially different patterns of both newly synthesized and accumulated major polypeptides [10], but at that time, it was not possible to obtain discrete sequences of these polypeptides, so their identity remained unknown.

A different approach, which is used in this study, is to investigate the different developmental programs in the two cell types by characterizing the abundance of novel or previously known mRNAs separately for each cell-type by real-time RT-PCR studies. This method should allow a considerable expansion of the number of genes investigated, studies done under different physiological conditions, and repeated experiments using mutant strains.

Here we show a quantitative analysis of a diversified pool of about forty target genes with regard to cell-type specific gene expression and relative expression rate in wild-type individuals of the green alga *Volvox carteri*. The investigated gene pool contains previously known genes with unknown localization, novel *Volvox *genes, which are described in this study for the first time, and a few previously characterized genes with known cell-type specific localization as controls. The corresponding gene products are, for instance, part of photosynthesis, cellular regulation, stress response, or transport processes.

## Results

### Target genes for differential analysis

Our goal was to investigate a diversified pool of target genes for cell-type specific expression. This pool contained both previously known genes with known or unknown localization and new genes (e.g. identified by homologs) with known, presumed, or unknown localization. The genes within this pool should vary both with respect to presumed function (if information about the function was available), e.g. genes related to photosynthesis, cellular regulation, stress response, or transport processes and also with respect to cellular localization (if any information about the localization was available). Specifically, a pool of 39 genes was built that contained the following 7 subsets of genes (Table 1):

**Table 1:** List of 39 *Volvox *genes and gene products, including subset classification, comparisons with homologs in other species, and significance of the sequence relationship to these homologs.

**Subset**	***V. carteri *gene** **(• novel *Volvox *gene)**	***V. carteri *gene product**	**Significance: percent identity with characterized homolog (# of identical residues/total # of residues)** **[similarity]**	**Gene product and species name of characterized homolog used for sequence comparison**
**A**	*actA*	actin [11] [GenBank:M33963]	98.0 % (370/377) [99.0 % (375/377)]	actin *Chlamydomonas reinhardtii *[55] [GenBank:D50839]
**A**	*ssgA*	pherophorin-like ECM-glycoprotein SSG185 [15] [GenBank:X51616]	54.4 % (137/252) [85.3 % (215/252)]	pherophorin-C3 *Chlamydomonas reinhardtii *[13] [GenBank:DQ196109]
**A**	*regA*	somatic regenerator RegA [9] [GenBank:AF106963]	67.5 % (81/120) [91.7 % (110/120)]	RegA-like sequence protein RlsA [9, 16] [GenBank:AF106962]
**A**	*gon30*	G30 protein [6, 56] [GenBank:AF110790]	85.4 % (380/445) [91.2 % (406/445)]	low-CO_2 _inducible protein LciC *Chlamydomonas reinhardtii *[43, 44] [GenBank:AB168094]
**A**	*gon167*	G167 protein [6] [GenBank:U31955]	99.1 % (210/212) [99.5 % (211/212)]	recombinase (ORF-C) on retrotransposon kangaroo-1 *Volvox carteri *[57] [GenBank:AY137241]
**B**	*rlsA*	RegA-like sequence protein RlsA [9, 16] [GenBank:AF106962]	67.5 % (81/120) [91.7 % (110/120)]	somatic regenerator RegA [9] [GenBank:AF106963]
**C**	*csrp1*	chloroplast-specific ribosomal protein [GenBank:AY835992]	36.7 % (79/215) [76.3 % (164/215)]	chloroplast-specific ribosomal protein PSrp-1, 30S subunit *Spinacia oleracea *[58] [GenBank:M55322]
**C**	*ard1*	C_2_H_2_-type zinc finger related protein with arsenite-resistance domain [GenBank:AY850006]	43.0 % (16/37) [62.0 % (23/37)]	RING finger protein 13 RNF13 *Gallus gallus *[GenBank:AY787020]
**C**	*mrp2*	ATP-energized ABC transporter Mrp2 [GenBank:AY835993]	91.4 % (243/266) [99.6 % (265/266)]	ABC transporter Mrp1 *Chlamydomonas reinhardtii *[59] [GenBank:AF442557]
**C**	*gspk47*	gonidia-specific protein KA_k47 [GenBank:AY835994]	---	---
**D**	*nitA*	nitrate reductase NitA [19] [GenBank:X64136]	80.8 % (698/864) [96.1 % (830/864)]	nitrate reductase Nit1 *Chlamydomonas reinhardtii *[60] [GenBank:AF203033]
**E**	*dyhA *•	flagellar α dynein (heavy chain) [GenBank: EF123072]	93.6 % (132/141) [97.8 % (138/141)]	flagellar α dynein (heavy chain) ODA11 *Chlamydomonas reinhardtii *[21, 22] [GenBank:L26049]
**E**	*klpA *•	kinesin-like protein [GenBank: EF123073]	84.8 % (117/138) [98.5 % (136/138)]	kinesin-like protein FLA10 *Chlamydomonas reinhardtii *[23] [GenBank:L33697]
**E**	*fer1 *•	ferredoxin Fer1 [GenBank: EF123074]	74.2 % (95/128) [93.7 % (120/128)]	ferredoxin PETF (chloroplast) *Chlamydomonas reinhardtii *[61] [GenBank:L10349]
**E**	*nab1 *•	nucleic acid binding protein Nab1 [GenBank: EF123075]	87.8 % (216/247) [97.1 % (239/247)]	nucleic acid binding protein Nab1 *Chlamydomonas reinhardtii *[25] [GenBank:AY157846]
**E**	*rap41 *•	ribosome-associated protein (chloroplast-specific) [GenBank: EF123076]	55.0 % (60/109) [89.9 % (98/109)]	ribosome-associated protein Rap41 (chloroplast-specific) *Chlamydomonas reinhardtii *[26] [GenBank:AY177616]
**E**	*fbp1 *•	chloroplast fructose-1,6-bisphosphatase [GenBank:EF123077]	61.1 % (196/318) [86.4 % (275/318)]	chloroplast fructose-1,6-bisphosphatase FBP *Brassica napus *[27] [GenBank:L15303]
**E**	*cp12 *•	Calvin cycle protein CP12 [GenBank: EF123078]	78.6 % (81/103) [97.1 % (100/103)]	Calvin cycle protein CP12 *Chlamydomonas reinhardtii *[28] [GenBank:AJ005284]
**F**	*prfA *•	profilin PrfA [GenBank: EF123079]	59.5 % (78/131) [90.8 % (119/131)]	profilin PRF1 *Chlamydomonas reinhardtii *[30] [GenBank:AF335423]
**F**	*fsd1 *•	superoxide dismutase Fsd1 [GenBank: EF123080]	88.7 % (204/230) [98.7 % (227/230)]	superoxide dismutase FSD1 *Chlamydomonas reinhardtii *[31] [GenBank:U22416]
**F**	*rpl37 *•	ribosomal protein L37 [GenBank: EF123081]	59.8 % (58/97) [87.6 % (85/97)]	ribosomal protein L37 (RPL37) *Homo sapiens *[32] [GenBank:NM_000997]
**F**	*glu1 *•	glutamate synthase [GenBank: EF123082]	68.4 % (575/841) [92.4 % (777/841)]	glutamate synthase GltB *Spinacia oleracea *[33] [GenBank:AF061515]
**F**	*hsp70B *•	heat shock protein 70 B [GenBank: EF123083]	70.9 % (141/199) [94.5 % (188/199)]	heat shock protein 70 Hsc70-7 *Arabidopsis thaliana *[34] [GenBank: AF217459]
**F**	*hsp40A *•	hsp40-like heat shock protein [GenBank: EF123084]	34.0 % (34/100) [70.0 % (70/100)]	heat-shock protein HSJ1 (DnaJ-like) *Homo sapiens *[35] [GenBank:NM_001039550]
**F**	*ubcA *•	ubiquitin conjugating enzyme E2 [GenBank: EF123085]	77.9 % (116/149) [94.0 % (140/149)]	ubiquitin conjugating enzyme E2 (UBC14) gene *Arabidopsis thaliana *[36] [GenBank:U33759]
**F**	*ponA *•	pontin [GenBank: EF123086]	76.7 % (345/450) [95.3 % (429/450)]	Pontin52 *Homo sapiens *[37] [GenBank: AF099084]
**F**	*mat3 *•	retinoblastoma-like protein Mat3 [GenBank: EF123087]	73.6 % (162/220) [92.3 % (203/220)]	retinoblastoma-like protein Mat3 *Chlamydomonas reinhardtii *[38] [GenBank: AF375824]
**F**	*vpeA *•	vacuolar processing enzyme VPE [GenBank: EF123088]	61.0 % (61/100) [86.0 % (86/100)]	vacuolar processing enzyme VPE-2 *Nicotiana tabacum *[39] [GenBank:AB075949]
**F**	*sac1 *•	sulfur deprivation response regulator Sac1 [GenBank: EF123089]	87.1 % (242/278) [98.6 % (274/278)]	sulfur deprivation response regulator Sac1 *Chlamydomonas reinhardtii *[40] [GenBank: U47541]
**F**	*rcd1 *•	required-for-cell-differentiation 1 protein Rcd1 [GenBank: EF123090]	69.5 % (196/282) [90.1 % (254/282)]	required-for-cell-differentiation 1 protein Rcd1 *Homo sapiens *[42] [GenBank:NM_005444]
**F**	*adcA *•	adenylate cyclase [GenBank:EF123091]	63.6 % (98/154) [86.4 % (133/154)]	adenylate cyclase, type II ADCY28 *Chlamydomonas reinhardtii *(JGI *Chlamydomonas *ID 121064)
**F**	*nipA *•	NaCl-inducible protein [GenBank: EF123092]	58.3 % (49/84) [86.9 % (73/84)]	NaCl-inducible protein (NIP) *Chlamydomonas reinhardtii *[GenBank:AU066522]
**F**	*lciB *•	low-CO_2 _inducible protein LciB [GenBank:EF123093]	56.3 % (190/339) [87.4 % (396/339)]	low-CO_2 _inducible protein LciB *Chlamydomonas reinhardtii *[43, 44] [GenBank:AB168093]
**G**	*upf1 *•	protein of unknown function [GenBank: EF123094]	---	---
**G**	*upf2 *•	protein of unknown function [GenBank:EF123095]	---	---
**G**	*upf3 *•	protein of unknown function [GenBank: EF123096]	---	---
**G**	*upf4 *•	protein of unknown function [GenBank: EF123097]	---	---
**G**	*upf5 *•	protein of unknown function [GenBank: EF123098]	---	---
**G**	*upf6 *•	protein of unknown function [GenBank: EF123099]	---	---

- Subset A: Known *Volvox *genes with known status of cell-type specific expression (5 genes: *actA*, *ssgA*, *regA*, *gon30*, *gon167*).

- Subset B: Known *Volvox *genes that have previously been identified via characterized homologs in *Volvox *and in which a cell-type specific expression is predictable due to the characteristics of the homologous gene (1 gene: *rlsA*).

- Subset C: Known *Volvox *genes with putative cell-type specific expression based on preliminary experiments (4 genes: *csrp1*, *ard1*, *mrp2*, *gspk47*).

- Subset D: Well-known *Volvox *genes with unknown status of cell-type specific expression (1 gene: *nitA*).

- Subset E: New *Volvox *genes that were identified in this project via characterized homologs in other species and in which a cell-type specific expression is predictable due to the characteristics of the homologs (7 genes: *dyhA, klpA*, *fer1*, *nab1*, *rap41*, *fbp1*, *cp12*).

- Subset F: New *Volvox *genes that were identified in this project via characterized homologs in other species but in which the status of cell-type specific expression is not predictable (15 genes: *prfA*, *fsd1*, *rpl37*, *glu1*, *hsp70B*, *hsp40A*, *ubcA*, *ponA*, *mat3*, *vpeA*, *sac1*, *rcd1*, *adcA*, *nipA*, *lciB*).

- Subset G: New *Volvox *genes for which no characterized homologs in any other organism have been identified and for which, consequently, the status of cell-type specific expression is unknown (6 genes: *upf1*, *upf2*, *upf3*, *upf4*, *upf5*, *upf6*).

### Characteristics of genes within subset A

Subset A contains 5 *Volvox *genes (Table 1). Each gene has been investigated previously for cell-type specific expression. The actin gene *actA*, expressed uniformly in somatic cells and gonidia [11], was used as a reference transcript in several previous studies [12-14] and is therefore used as the reference gene in the real-time experiments described below. The gene coding for the extracellular matrix glycoprotein SSG185, *ssgA*, is known to be expressed mainly by somatic cells [15]. Similarly, the *regA *gene, a key gene controlling cell differentiation in *Volvox carteri *by suppressing reproductive activities in somatic cells, is expressed only in somatic cells [9]. In a previously described search for cell-type specific genes of *Volvox carteri *[6], several gonidia-specific genes have been identified. Two of these genes, *gon30 *and *gon167*, are included in our study. The *gon167 *mRNA has been shown to be present in variable but moderate levels in gonidia, with highest expression levels at the beginning of cleavage and during cleavage divisions. In contrast, the *gon30 *mRNA was at its lowest during cell cleavages and had maximal expression much later. For both *gon30 *and *gon167*, the mRNA level in somatic cells has been shown to be low at all stages.

### Characteristics of genes within subset B

Subset B contains only one *Volvox *gene (Table 1), the *rlsA *gene, which has previously been identified via the homologous *Volvox *gene *regA *[9,16]. Partial sequences of *rlsA *and *regA *show extensive similarity, including fully conserved exon/intron boundaries. Since *regA *is expressed only in somatic cells (see above), a cell-type specific expression of *rlsA *in somatic cells seems to be probable, but cell-type specific expression has not been investigated so far.

### Characteristics of genes within subset C

Subset C contains 4 *Volvox *genes (Table 1) that have been identified by differential screenings of cDNA libraries of gonidia versus somatic cells in the group of Dr. R. Schmitt (University of Regensburg, Germany) [17,18] and which have been deposited in GenBank, but experimental details are not available. Two of these genes are described as specific for somatic cells: a gene coding for a putative chloroplast-specific ribosome-associated protein (*csrp1*, "KSS_k11") and one that codes for a putative arsenite-resistance protein (*ard1*, "KSS_k05"). Both remaining genes are described as specific for gonidia: a gene coding for a putative ATP-energized ABC transporter (*mrp2*, "KA_k18/MH_k18") and one that codes for a putative gonidia-specific protein (*gspk47*, "KA_k47").

### Characteristics of genes within subset D

Only one gene is within subset D (Table 1). It is the gene that codes for nitrate reductase, *nitA *[19], which has previously been characterized in much detail. This gene is also the standard selectable marker in *Volvox *transformation experiments [20]. Nevertheless, to our knowledge, this well-investigated gene has never been analyzed for cell-type specific expression in *Volvox*.

### Characteristics of genes within subset E (novel *Volvox *genes)

**Figure 2:**
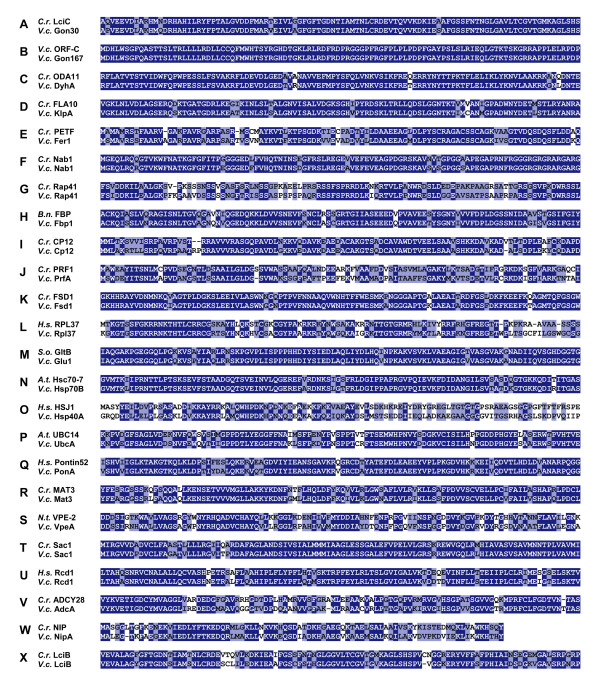
**Pairwise sequence alignment of amino acid sequences**. Alignment of sequences, deduced from *Volvox *genes used in this study, with previously known proteins characterized in other species. Identical residues are given as white letters on a dark blue background. Similar residues are given as black letters on a light blue background. The sequence alignment was done using DNASIS/PROSIS Software. With the exception of NIP/NipA, which are shorter, a section of 100 residues is shown. Species names: *A.t*., *Arabidopsis thaliana*; *B.n*., *Brassica napus*; *C.r*., *Chlamydomonas reinhardtii*; *H.s*., *Homo sapiens*; *N.t*., *Nicotiana tabacum*; *S.o*., *Spinacia oleracea*; *V.c*., *Volvox carteri*.

The seven genes within subset E (Table 1) were obtained by searching the *Volvox *whole-genome shotgun reads at the *Chlamydomonas *web site of the Joint Genome Institute with sequences of well-known genes from other species. The significance of the sequence relationship is illustrated by a 100 residue long part of the sequence alignment (Fig. 2) and indication of percent identity and similarity (Table 1). A cell-type specific expression of these novel *Volvox *genes seems to suggest itself due to the characteristics of the homologs. Two of these seven genes, *dyhA *and *klpA*, were predicted to be expressed in somatic cells. *dyhA*, is a homolog of *oda11 *[21,22], a *C. reinhardtii *gene coding for the flagellar outer row dynein alpha heavy chain. *klpA *codes for a kinesin-like protein that is involved in motility associated with the flagellar membrane [23] and has been shown to be localized to the region between the axonemal outer doublet microtubules and the flagellar membrane in the unicell *C. reinhardtii *[24]. Because only somatic *V. carteri *cells have flagella, a cell-type specific expression of *dyhA *and *klpA *in somatic cells can be expected.

The other five genes were predicted to be expressed only in the gonidia. They are nuclear genes that encode for chloroplast-targeted proteins. *fer1 *encodes a ferredoxin; this protein family contains Fe-S clusters which plays a key role in electron-transfer during photosynthesis. *nab1 *codes for an RNA binding protein involved in the light-regulated differential expression of the light-harvesting antenna [25]. *rap41 *codes a ribosome-associated protein, which has been identified only in the 70S ribosome of the chloroplast [26]. *fbp1 *encodes a chloroplast-specific fructose-1,6-bisphosphatase, a key enzyme of the Calvin cycle for photosynthetic CO_2 _assimilation [27]. *cp12 *codes for Calvin cycle protein CP12, a small chloroplast protein, which is essential for the assembly of the phosphoribulokinase/glyceraldehyde-3-phosphate dehydrogenase (GAPDH) complex [28]. In *V. carteri*, major metabolic activities of the chloroplast are encoded by nuclear genes that are attenuated in mature somatic cells [29], which is why the small chloroplasts of somatic cells don't grow in size. Therefore, photosynthesis-related nuclear genes within subset E are expected to be expressed mainly within the gonidia.

### Characteristics of genes within subset F (novel *Volvox *genes)

As in subset E, all fifteen genes within subset F (Table 1) were obtained by searching the *Volvox *whole-genome shotgun reads at the *Chlamydomonas *web site of the Joint Genome Institute with sequences of well-known genes from other species (Fig. 2). In contrast to subset E, there is no concrete information or indication about cell-type specific expression for any of these genes. The novel *Volvox *genes within subset F code for the following proteins (gene names in parentheses): profilin (*prfA*), an actin binding protein [30]; superoxide dismutase (*fsd1*), a key enzyme in the antioxidant defense system [31]; ribosomal protein L37 (*rpl37*), a 60S ribosomal subunit protein [32]; glutamate synthase (*glu1*), an enzyme that catalyzes the reductive amination of α-ketoglutarate [33]; heat shock protein 70B (*hsp70B*), which is involved in folding of nascent polypeptides [34]; an hsp40-like heat shock protein (*hsp40A*), which might be a cochaperone protein that regulates complex formation between Hsp70 and client proteins [35]; a ubiquitin conjugating enzyme E2 (*ubcA*), which controls specificity of ubiquitin-protein conjugation during selective protein degradation [36]; pontin (*ponA*), a transcriptional cofactor which is known to play an essential role in the control of cellular growth and proliferation during development [37]; a retinoblastoma-like protein (*mat3*), which is involved in the control of cell division in *C. reinhardtii *[38]; vacuolar processing enzyme (*vpeA*), a vacuolar protease with caspase-1 activity, which is involved in programmed cell death in higher plants [39]; a sulfur deprivation response regulator (*sac1*), a protein which is critical for survival of *C. reinhardtii *during sulfur deprivation and which is involved in control of cysteine biosynthesis [40,41]; a required-for-cell-differentiation 1 protein (*rcd1*), which is a transcriptional cofactor that mediates retinoic acid-induced cell differentiation in *Homo sapiens *[42]; adenylate cyclase (*adcA*), the enzyme that catalyzes the conversion of ATP to 3',5'-cyclic AMP and pyrophosphate; an NaCl-inducible protein (*nipA*), a very small protein that has been identified in the halotolerant *C. reinhardtii *strain HS-5; and finally, a low-CO_2 _inducible protein LciB (*lciB*), which seems to be part of a carbon-concentrating mechanism in *C. reinhardtii *[43,44].

### Characteristics of genes within subset G (novel *Volvox *genes)

Subset G contains 6 *Volvox *genes with unknown function and unknown localization (Table 1). Though we found homologs of these genes in other species, especially in *C. reinhardtii*, none of the homologous genes or gene products has previously been characterized. We named these genes *upf1*, *upf2*, *upf3*, *upf4*, *upf5*, and *upf6*.

### Selection of primer combinations and testing by genomic PCR and RT-PCR

Oligonucleotide primers have been designed for the selected 39 *Volvox *genes for real-time RT-PCR experiments. cDNA amplicons were planned to be only 100–160 bp in size, which is an optimal size for real-time RT-PCRs. When possible, primers were designed in two successive exons in order to uncover a potential contamination with genomic DNA, since such a product would be larger than expected due to the intron in between. Exon-intron prediction of *Volvox *genes was done using FGENESH software (Softberry, Mount Kisco, NY) and by polypeptide sequence comparisons with homologs from other organisms using PROSIS software (version 7.00; Hitachi Software Engineering, South San Francisco, CA). Before utilization in real-time RT-PCR experiments, all primers were tested by genomic PCR for their specificity and reproducibility using total genomic DNA as a template. Simultaneously, the amplification conditions were optimized (data not shown). When necessary, unsuitable primers, which produced either the wrong product, a weak product, or more than one product, were replaced, and the procedure was repeated. Similarly, the primers were also tested by standard RT-PCR, using total RNA as template, in order to prove the quality of the oligonucleotide primers (data not shown). Primers that met all requirements, i.e. there is only a single distinct amplicon of the predicted size, are given in Table 2, and the sizes of cDNAs and genomic DNAs (gDNAs) are listed as well.

**Table 2:** Primers used for quantitative real-time RT-PCR and lengths of cDNA and gDNA products.

**Name**	**Forward Primer (5'→3')**	**Reverse Primer (5'→3')**	**Product length** **cDNA**	**Product length** **gDNA**
*actA*	TGAGAAGACGTACGAGCTGC	CCTCCATGCCGATTAGGCTA	104	241
*ssgA*	TTCGCATCGTGAAGGACCTT	CCGTTAACGTCCATGAACAG	130	780
*regA*	CAATGGCAGCAAATGGATGTC	GTTCCAAATCAGGCAACACG	101	1339
*gon30*	CCATGTTTGTGTCCTCTCCA	GGTTATCGAGGCAGGCATTA	106	106
*gon167*	GCTTCTATCGTTTGCGGAAG	GCACGCATACAACCTACAC	112	112
*rlsA*	CACAATGGCAGCAAATGGATG	GGTTCCAAATCAGGCAACAC	104	1445
*csrp1*	TGTGTGACTCCTGCAAGCA	TGGATGATGATGCGAACGG	149	266
*ard1*	TGGCTAGGCCATATCCTTTG	TCGATGGTGTTCCTACGTGA	144	144
*mrp2*	GTGGTTCGTTTCATCGCTG	TGCATCCAGACATCCTCGA	125	519
*gspk47*	ATTCGACAAGGTGGACAAGG	GGTCTTGACAGGGATAGGA	116	116
*nitA*	CCACGAGCACTACCTACTG	CCGGAAGCACACGAAGTTG	101	584
*dyhA*	AACGCTGTCGTGGAGTTCAT	TTCGGGGTCGTGTAGTTGT	101	302
*klpA*	GCCACCAAGATTAACCTGTC	CGCACATGATTGTCTTCGTG	151	908
*fer1*	ATGGACCTGCCGTACTCTTG	GCCTCCATCTGCTTATCGTC	116	116
*nab1*	GGAACCGTGAAGTGGTTCAAC	CCTCAACCTCAAACTCGACG	122	498
*rap41*	TCTCCACAAGGCTCTAGTTG	GTGTAGATGGTCGCGATATC	123	123
*fbp1*	GGTGAGGACCAGAAGAAGC	GAGTAGGTCTCCTCCACAG	131	453
*cp12*	AAGTACTGCCAGGACGCTC	GCTCGTCGTGACGCAGTAA	129	289
*prfA*	ACGCCTGAAGAGTTCGAGA	GCAGAACAGTCTCGTCAGA	112	112
*fsd1*	AGATCGTCCTGGCTAGCTG	CACCATTTGCCTTCATGCTTTC	146	146
*rpl37*	ACCAGCTACCACAACCAGAA	AAGCGTCTTCATGTACCGCA	138	345
*glu1*	AGGCTATGACTAAGCTCGAG	ATCGCCTTATGCAGCAGCTT	103	295
*hsp70B*	AGGTCTTTTCCACTGCTGCC	TCAATTTGTGGAACTCCACGCG	148	454
*hsp40A*	GCCAGGGATGATGTTCAACT	TTAACGATGCGTGCACTCCT	130	130
*ubcA*	GGTTTCTTCAACGCAAAGCTA	ATGGAGGATGGAAATGCAGA	129	198
*ponA*	AAGAAGACGGAGATCACGGA	GTGAAGCACTCGATATCCAG	140	1029
*mat3*	GCAGATAGTGCTTAAGCTTGG	CGTGACTAACGAGGATGGC	128	274
*vpeA*	CACTGGGCTCTGCTAGTAG	CTATGTCGTCGTACATCATGAC	139	560
*sac1*	GTCACCGGTGTACTTACCGTA	CATCCACTTGAAGCAGCTCA	155	633
*rcd1*	GTGCGTGGCGTCTCACAATG	CAACCGCAGGTACTCGAAGG	118	260
*adcA*	GTGCCATATGTCGGATTTCTG	CATGACGATCACGACGTTTC	144	144
*nipA*	GGGTGAGAAAGCGATTGAAG	TTGTCCGCAATGTCCGACT	102	373
*lciB*	CCGTCGACGACTTTATCTCC	AGACTCATCTCGGCACAGGT	101	438
*upf1*	CTTTGAGCTGCTGCAACACC	GGAGCGTGTGACCTACTG	152	914
*upf2*	GTTGCCGCCATGGATTTCC	GGTTGTTAAGCGCAACACGTA	113	113
*upf3*	CGTTATGGCTGCATATCACC	ACATTTCATAACCGAACAACACCAC	108	108
*upf4*	AAATTGCATCGCTGCAAGCG	AAAACCGGCAAGTGTCACTC	133	133
*upf5*	CAGTTAACGGCTCAACATTGG	CATGCACGTAAGCTTTCTTCC	128	128
*upf6*	TGCACGAGAGCTGTTGGTT	CACGCCTTAGTGCGAATATC	135	135

### Analysis of cell-type specific gene expression by real-time RT-PCR experiments

*Volvox *spheroids were broken in a Dounce homogenizer, and cell-size based separation of cell types was achieved by successive filtration on screens of different mesh sizes and a centrifugation step (Fig. 1B, C); total RNA was isolated from the separated cell types using the phenol-based TRI Reagent (see Methods).

**Figure 3:**
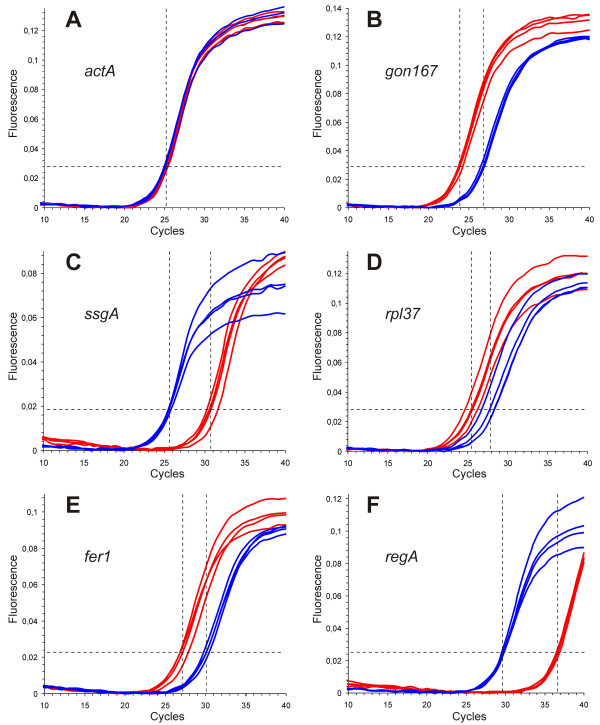
**Comparison of gene expression of six target genes in gonidia versus somatic cells by quantitative real-time RT-PCR**. Amplification curves for A) *actA *(internal control for the 2^-ΔΔCt ^method), B) *gon167*, C) *ssgA*, D) *rpl37*, E) *fer1*, and F) *regA*. The target-specific fluorescence signal of SYBR Green fluorescence emission (detection range 515–545 nm) is plotted against the number of PCR cycles. Curves of gonidial RT-PCRs are given in red, somatic RT-PCRs in blue. All real-time RT-PCR experiments were carried out in triplicate, and a mean amplification curve was generated for each cell-type. The threshold level is given by a broken, horizontal line. The cycle at which the mean amplification curve of gonidial or somatic real-time RT-PCRs crosses the threshold (C_t _value) is indicated by a broken, vertical line.

**Figure 4:**
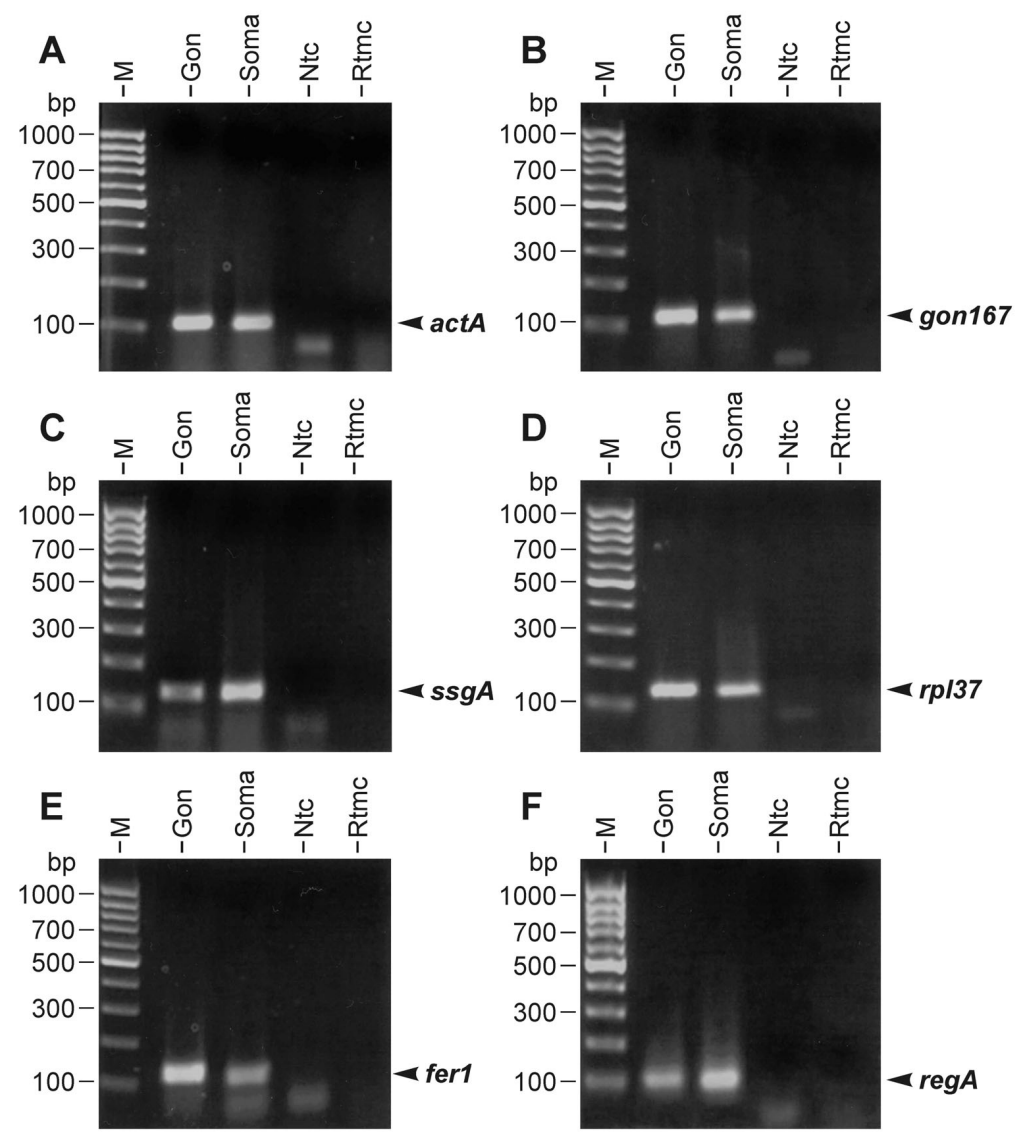
**Visualization of final products after real-time RT-PCRs of six target genes**. The following sizes have been predicted for the amplified cDNA fragments of the corresponding mRNAs: A) *actA*, 104 bp; B) *gon167*, 112 bp; C) *ssgA*, 130 bp; D) *rpl37*, 138 bp; E) *fer1*, 116 bp; and F) *regA*, 101 bp. RT minus and no template controls were free of any DNA product as expected. The lanes on the agarose gels were loaded with: M, 100 bp size marker; Gon, reaction product from gonidia; Soma, reaction product from somatic cells; Ntc, no template control; Rtmc, RT minus control.

**Figure 5:**
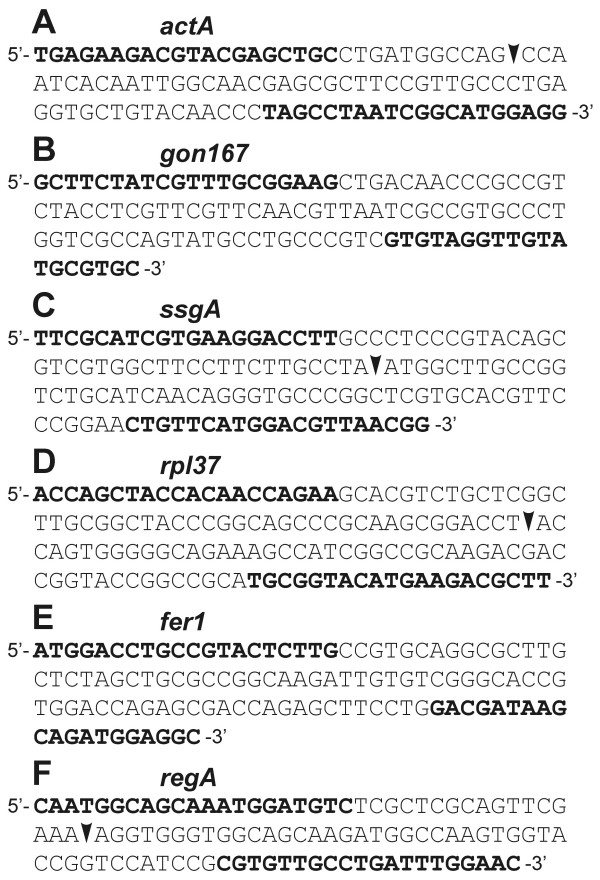
**Sequencing of real-time RT-PCR products of six target genes**. A) *actA*, B) *gon167*, C) *ssgA*, D) *rpl37*, E) *fer1*, and F) *regA*. The positions of PCR primers are indicated in bold. Positions of introns within these cDNA-fragments are indicated by arrowheads. All mRNAs have been spliced as predicted, and the cDNA fragments, which have been obtained by the above mentioned real-time RT-PCRs, showed the expected sequences.

**Figure 6:**
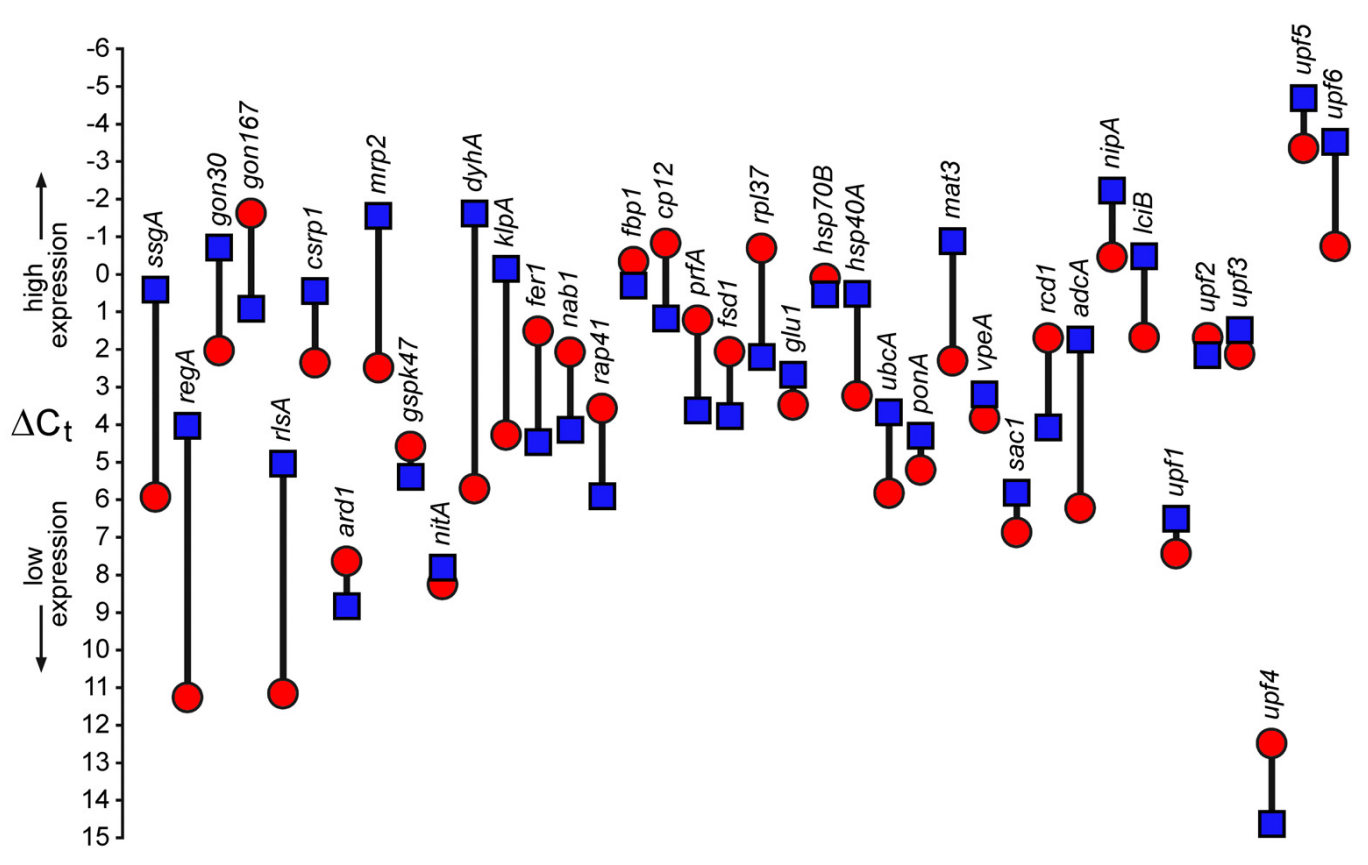
**Visualization of ΔC_t _values of 38 genes from *V. carteri *somatic cells and gonidia**. Blue quadrangles: values from somatic cells; red circles: values from gonidia. Each pair of ΔC_t _values for a single gene is connected by a vertical black bar; the length of this bar corresponds to the ΔΔC_t _value. *actA *was used as a reference and thus defines the zero line.

**Figure 7:**
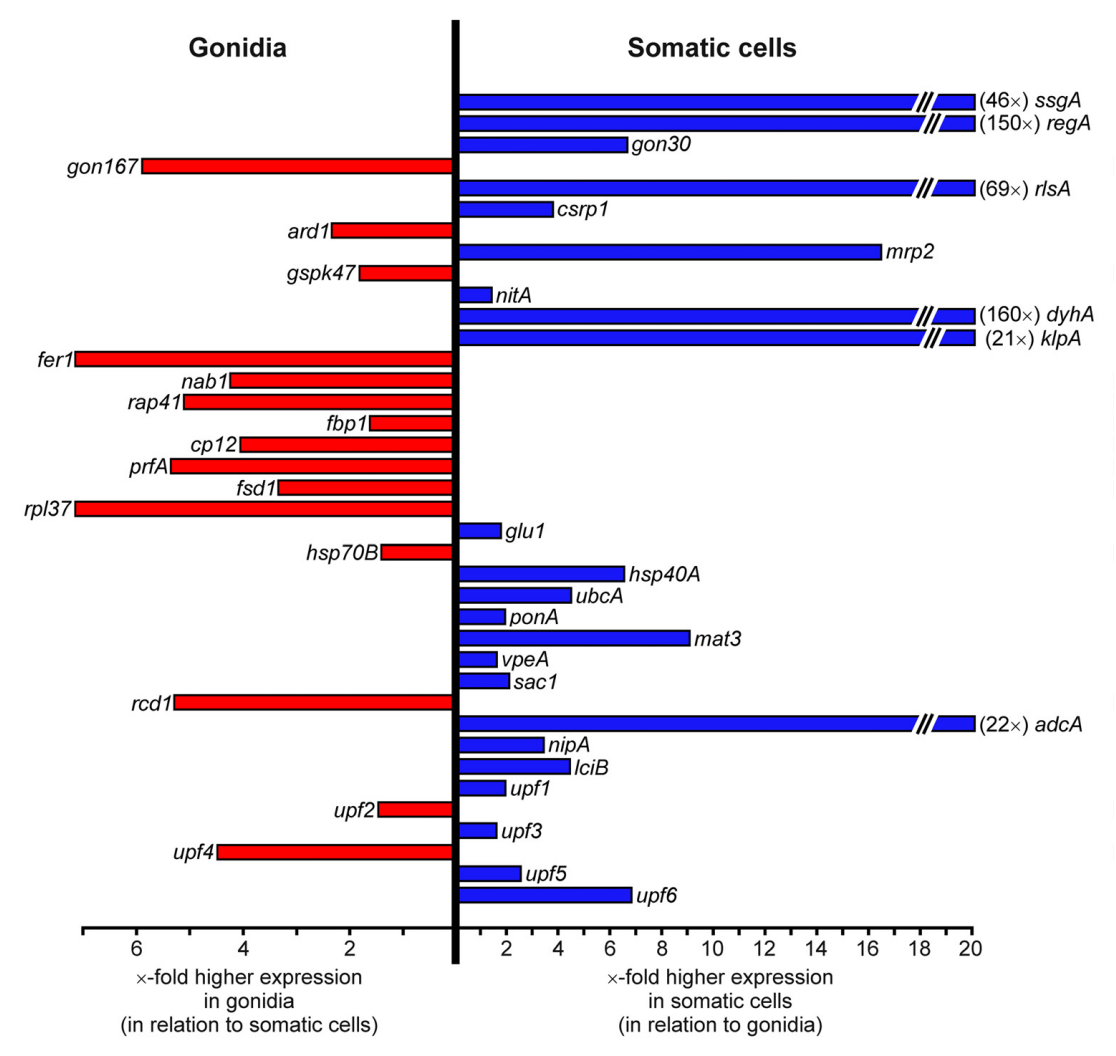
**Cell-type specific gene expression of 38 genes from *V. carteri *determined by the 2^-ΔΔCt ^method**. The gene names are given at the end of the horizontal expression bars. The length of the expression bar illustrates the ×-fold higher expression of a given target gene within the given cell type with respect to the other cell type. Blue: higher expression in somatic cells; red: higher expression in gonidia.

Real-time RT-PCR reactions were performed using the selected primers (Table 2) and a DNA Engine Opticon Continuous Fluorescence Detection System (see Methods). All real-time RT-PCR experiments have been carried out in triplicate together with RT minus and no template controls. The set of 39 genes was investigated separately both in somatic cells and gonidia by using identical amounts of RNA. For the sake of accuracy and precision, it is necessary to collect quantitative data at a point in which every sample is in the exponential phase of amplification, since it is only in this phase that amplification is extremely reproducible. By way of example, the amplification curves of 6 genes, *actA*, *gon167*, *ssgA*, *rpl37*, *fer1 *and *regA*, are illustrated in Figure 3. To detect and exclude non-specific amplicons, the melting curves of all PCR products have been analyzed (data not shown); moreover, all final products have been investigated for multiple bands on agarose gels; as an example, the result for the above mentioned 6 genes is shown in Figure 4. All obtained fragment sizes have been compared to the calculated fragment sizes (Table 2). Needless to say that RT minus and no template controls always had to be free of any DNA product in order to exclude the possibility of the amplification of genomic DNA. A second primer pair has been chosen for any gene of interest that had one ambiguous criterion. Real-time RT-PCR products with correct sizes have also been verified by sequencing (Fig. 5). Results from real-time PCR experiments that finally met all these requirements are shown in Table 3. All real-time RT-PCR experiments have been carried out in triplicate together with RT minus and no template controls (see Methods). The results were represented as cycle threshold (C_t_) values. There is an inverse correlation between C_t _values and the amount of target mRNA: higher amounts of target mRNA have lower C_t _values, and lower amounts of target mRNA correspond to a higher C_t _value. ΔC_t _was determined as the mean of the triplicate C_t _values for the target genes minus the mean of the triplicate C_t _values for the actin gene. ΔΔC_t _represented the difference between the two cell types for a given target gene, more precisely ΔΔC_t _= ΔC_t (gonidia) _- ΔC_t (somaticcells)_. Standard deviations for all ΔC_t _and ΔΔC_t _values (gonidia versus somatic cells) are given within Table 3. To allow a better overview and to show the wide spectrum of expression rates, all ΔC_t _and ΔΔC_t _values (without standard deviations) are illustrated within a single figure (Fig. 6). *actA *was used as a reference, therefore 38 (not 39) genes are shown. The actual expression level of a given target gene in one cell type versus the other cell type was analyzed using the 2^-ΔΔCt ^method (see Methods). The results, including standard deviations, are listed in Table 3, and the 2^-ΔΔCt ^values are illustrated in Figure 7. The calculation results in 15 genes that are higher expressed in gonidia than in somatic cells and 23 genes that are higher expressed in somatic cells than in gonidia. However, 10 of these genes show less than a two-fold difference in the expression rate, namely *gspk47*, *nitA*, *fbp1*, *glu1*, *hsp70B*, *ponA*, *vpeA*, *upf1*, *upf2*, and *upf3*, so the difference between the two cell types is only minor or even insignificant. With two additional genes, *csrp1 *and *ard1*, the standard deviation is higher than the difference in expression rate, so the classification of both genes is questionable. Consequently, 10 genes remain which show an explicitly higher expression rate in gonidia than in somatic cells (*gon167*, *fer1*, *nab1*, *rap41*, *cp12*, *prfA*, *fsd1*, *rpl37*, *rcd1*, and *upf4*), and another 16 genes demonstrate a definitively higher expression rate in somatic cells than in gonidia (*ssgA*, *regA*, *gon30*, *rlsA*, *mrp2*, *dyhA*, *klpA*, *hsp40A*, *ubcA*, *mat3*, *sac1*, *adcA*, *nipA*, *lciB*, *upf5*, and *upf6*).

**Table 3:** Results of real-time RT-PCR experiments. Standard deviations are given in parentheses.

**mDNA**	**ΔC_t _gonidia**	**ΔC_t _somatic cells**	**ΔΔC_t_**	**2^-ΔΔCt ^or 1/2^-ΔΔCt^**	×**-fold higher expression in**
*ssgA*	5.94 (± 0.7)	0.42 (± 0.2)	5.52 (± 0.8)	45.97 ×	somatic cells
*regA*	11.27 (± 0.3)	4.04 (± 0.2)	7.23 (± 0.2)	149.72 ×	somatic cells
*gon30*	2.04 (± 0.5)	-0.69 (± 0.5)	2.73 (± 0.3)	6.62 ×	somatic cells
*gon167*	-1.62 (± 0.2)	0.92 (± 0.3)	-2.54 (± 0.2)	5.82 ×	gonidia
*rlsA*	11.17 (± 0.8)	5.06 (± 0.5)	6.12 (± 0.4)	69.32 ×	somatic cells
*csrp1*	2.36 (± 1.7)	0.46 (± 0.2)	1.90 (± 2.0)	3.74 ×	somatic cells
*ard1*	7.65 (± 0.6)	8.84 (± 0.8)	-1.18 (± 1.4)	2.27 ×	gonidia
*mrp2*	2.49 (± 0.1)	-1.55 (± 0.7)	4.04 (± 0.6)	16.45 ×	somatic cells
*gspk47*	4.59 (± 0.3)	5.40 (± 0.2)	-0.81 (± 0.4)	1.75 ×	gonidia
*nitA*	8.27 (± 0.3)	7.82 (± 0.6)	0.45 (± 0.3)	1.36 ×	somatic cells
*dyhA*	5.71 (± 0.2)	-1.61 (± 0.2)	7.32 (± 0.4)	159.82 ×	somatic cells
*klpA*	4.29 (± 0.6)	-0.13 (± 0.4)	4.42 (± 0.2)	21.42 ×	somatic cells
*fer1*	1.52 (± 0.0)	4.47 (± 0.3)	-2.95 (± 0.2)	7.73 ×	gonidia
*nab1*	2.08 (± 0.4)	4.14 (± 0.0)	-2.06 (± 0.1)	4.17 ×	gonidia
*rap41*	3.58 (± 0.0)	5.92 (± 0.4)	-2.33 (± 0.4)	5.04 ×	gonidia
*fbp1*	-0.33 (± 0.4)	0.31 (± 0.2)	-0.64 (± 0.5)	1.56 ×	gonidia
*cp12*	-0.82 (± 0.2)	1.18 (± 0.2)	-1.99 (± 0.1)	3.98 ×	gonidia
*prfA*	1.23 (± 0.5)	3.63 (± 0.1)	-2.40 (± 0.4)	5.29 ×	gonidia
*fsd1*	2.07 (± 0.2)	3.78 (± 0.4)	-1.71 (± 0.5)	3.27 ×	gonidia
*rpl37*	-0.68 (± 0.5)	2.21 (± 0.0)	-2.89 (± 0.5)	7.43 ×	gonidia
*glu1*	3.49 (± 0.2)	2.70 (± 0.2)	0.79 (± 0.3)	1.73 ×	somatic cells
*hsp70B*	0.12 (± 0.3)	0.54 (± 0.0)	-0.43 (± 0.4)	1.34 ×	gonidia
*hsp40A*	3.24 (± 0.4)	0.54 (± 0.3)	2.70 (± 0.2)	6.50 ×	somatic cells
*ubcA*	5.84 (± 0.0)	3.69 (± 0.1)	2.15 (± 0.0)	4.44 ×	somatic cells
*ponA*	5.22 (± 0.5)	4.31 (± 0.2)	0.91 (± 0.3)	1.88 ×	somatic cells
*mat3*	2.31 (± 0.1)	-0.87 (± 0.2)	3.18 (± 0.1)	9.03 ×	somatic cells
*vpeA*	3.84 (± 0.2)	3.20 (± 0.5)	0.64 (± 0.3)	1.56 ×	somatic cells
*sac1*	6.88 (± 0.1)	5.84 (± 0.1)	1.03 (± 0.2)	2.04 ×	somatic cells
*rcd1*	1.70 (± 0.6)	4.08 (± 0.1)	-2.38 (± 0.7)	5.22 ×	gonidia
*adcA*	6.23 (± 0.3)	1.76 (± 0.6)	4.47 (± 0.4)	22.18 ×	somatic cells
*nipA*	-0.45 (± 0.1)	-2.21 (± 0.4)	1.76 (± 0.4)	3.39 ×	somatic cells
*lciB*	1.68 (± 0.5)	-0.46 (± 0.6)	2.14 (± 0.1)	4.40 ×	somatic cells
*upf1*	7.44 (± 0.4)	6.51 (± 0.2)	0.93 (± 0.6)	1.90 ×	somatic cells
*upf2*	1.69 (± 0.1)	2.16 (± 0.2)	-0.47 (± 0.3)	1.39 ×	gonidia
*upf3*	2.14 (± 0.4)	1.50 (± 0.1)	0.64 (± 0.6)	1.55 ×	somatic cells
*upf4*	12.50 (± 0.2)	14.64 (± 0.7)	-2.14 (± 0.9)	4.41 ×	gonidia
*upf5*	-3.35 (± 0.1)	-4.67 (± 0.1)	1.32 (± 0.2)	2.49 ×	somatic cells
*upf6*	-0.74 (± 0.4)	-3.51 (± 0.1)	2.76 (± 0.3)	6.78 ×	somatic cells

The greatest difference in expression rate between both cell types was seen in *dyhA *which reveals a ~160-fold higher expression in somatic cells than in gonidia.

## Discussion

A quantitative analysis of cell-type specific gene expression by real-time RT-PCR requires a quick, efficient, and quantitative method for the physical removal of one cell type from another. After mechanical disruption of a multicellular organism, existing separation methods in other species take advantage of cell size, density, surface charge, hydrophobic surface properties, and antigen status in order to separate cell types. These methods are quite complicated in the majority of cases and include sedimentation, centrifugal elutriation, partitioning in aqueous two-phase systems, flow cytometry, immuno methods (including magnetic, column, and panning techniques), and free flow electrophoresis [45]. In contrast, viable *Volvox *cells can be separated quite easily (see Methods) (Fig. 1B, C).

To identify divergent transcriptional activities in the two morphologically and functionally distinct cell types of *Volvox carteri*, we analyzed a pool of 39 genes; these genes were grouped into 7 subsets.

### Gene subsets A-C: comparison of the obtained real-time RT-PCR results with the expectations

Four out of five mRNAs of subset A were localized just as expected: *actA *was expressed uniformly in somatic cells and gonidia and was used as a reference [11]. *ssgA *and *regA *were expressed mainly in somatic cells, as previously described [9,15]; *ssgA *showed a ~46-fold higher expression in somatic cells than in gonidia and expression of *regA *was ~150-fold higher in somatic cells. The gonidia-specific gene *gon167 *[6] showed a ~6-fold higher expression in gonidia, just as expected. Only the gonidia-specific gene *gon30 *[6] didn't meet the expectations at first sight, since there was a higher expression in somatic cells as compared to gonidia. However, *gon30 *is a very late "gonidial" gene [6], which has its maximal expression after cell cleavages, and the mRNAs for our experiments were isolated at the very beginning of cell cleavages. In contrast to *gon30*, expression of *gon167 *peaks at the beginning of cleavages. In the case of *gon30*, we obviously compared only the minimal mRNA level in gonidia with that of somatic cells; at this point, expression of *gon30 *is higher in somatic cells than in gonidia.

The mRNA of the sole member of subset B, *rlsA*, was localized as expected. Similar to the homologous gene *regA*, the *rlsA *gene is strongly expressed in somatic cells; there was a ~69-fold higher expression in somatic cells than in gonidia.

Subset C contained 4 genes with putative cell-type specific expression based on preliminary experiments [17,18]. As expected, *csrp1 *was expressed mainly in somatic cells, but our result with this particular gene was not so clear due to high standard deviations for the ΔC_t _and ΔΔC_t _values (see above). *gspk47 *was expressed mainly in gonidia, as expected, although the ΔΔC_t _value was rather low. We couldn't confirm the localization of *ard1 *and *mrp2*: in our hands *ard1 *was expressed mainly in gonidia, and *mrp2 *was expressed mainly in somatic cells, with a ~16-fold higher expression in somatic cells. These results contradict previous preliminary results. The discrepancies between earlier results and our results within subset C might follow from differences in the experimental approach: we used a wild-type *Volvox carteri *strain for preparation of both gonidia as well as somatic cells. In the group of Dr. R.Schmitt [17,18] a wild-type strain was used only for preparation of somatic cells, whereas the gonidia were isolated from a *regA*-mutant strain in which the somatic cells de-differentiated to gonidia because in this way it is much easier to isolate large amounts of RNA from this cell type. In the light of our results, it seems questionable that these secondary gonidia of *regA*-mutants show exactly the same expression pattern as wild-type gonidia. Another problem might be that it is not possible to synchronize *regA*-mutants (in contrast to wild-type algae); therefore every RNA preparation from *regA*-mutants is more or less heterogeneous with respect to the developmental stage and might even contain RNA from somatic cells that have not begun to de-differentiate.

### Gene subsets D-G: validation of the obtained real-time RT-PCR results

Subset D contained only a single, well-known *Volvox *gene with a previously unknown status of cell-type specific expression. Here we show that this gene, *nitA*, is expressed more or less uniformly in somatic cells and gonidia (there is only an insignificant, 1.36-fold higher expression in somatic cells). The gene product of *nitA*, nitrate reductase, plays a central role in nitrate acquisition because it is the first enzyme in the pathway, and it is required for growth when nitrate is the sole nitrogen source. Therefore, it makes sense that both cell types express this gene similarly.

Subset E covered 7 novel *Volvox *genes, *dyhA*, *klpA*, *fer1*, *nab1*, *rap41*, *fbp1*, and *cp12*, which were identified via their characterized homologs from other species and in which, therefore, a cell-type specific expression seemed to be predictable due to the characteristics of the homologs. The motility-related genes *dyhA *and *klpA *showed, respectively, a ~160-fold and ~21-fold higher expression in somatic cells. This is a reasonable result because in *V. carteri *only somatic cells have flagella. Likewise, all five putative chloroplast/photosynthesis-related *Volvox *genes, *fer1*, *nab1*, *rap41*, *fbp1*, and *cp12*, were shown to be expressed predominantly in gonidia. Since chloroplast/photosynthesis-related metabolic activities are known to be localized mainly in the huge chloroplasts of gonidia and only to a minor degree in the small chloroplasts of somatic cells, the obtained expression rates are logical.

All 15 new *Volvox *genes within subset F have been identified via characterized homologs in other species, but, in contrast to the genes within subset E, the status of cell-type specific expression of these genes was not predictable. Four genes, *prfA*, *fsd1*, *rpl37*, and *rcd1*, were shown to be expressed predominantly in gonidia, and another seven genes, *hsp40A*, *ubcA*, *mat3*, *sac1*, *adcA*, *nipA*, and *lciB*, were mainly expressed in the somatic cells. The last four genes, *hsp70B*, *glu1*, *vpeA*, and *ponA*, were more or less uniformly expressed in somatic cells and gonidia; *hsp70B *showed a slightly higher expression level in gonidia, whereas *glu1*, *vpeA*, and *ponA *expression was somewhat higher in somatic cells.

Within subset G, which contains 6 new *Volvox *genes for which no characterized homologs in any other organism have been identified and the status of cell-type specific expression was unknown, one gene, *upf4*, was mainly expressed in gonidia, and two others, *upf5 *and *upf6*, were shown to be expressed predominantly in somatic cells. *upf2 *showed only a somewhat higher expression in gonidia, and expression of *upf1 *and *upf3 *was just slightly higher in somatic cells; these three genes were more or less uniformly expressed in the different cell types. Future experiments will have to reveal the concrete functions of the genes within subset G.

Taking into account all the gene subsets, it can be stated that if a prediction for expression in a specific cell-type was possible, this prediction came true, except for two genes within subset C. However, the discrepancy within subset C can possibly be explained by differences in the experimental approach (see above). Our findings with respect to the *gon30 *gene serve as a warning to any future investigators who employ these methods that it will be important to pay not only close attention to the spatial aspects of differential gene expression but also to temporal aspects; in addition, environmental conditions (light, temperature, culture medium etc.) should be kept in mind.

As expected, gonidia and somatic cells clearly differ in the composition of their mRNA pools, since it is this difference in cell-type specific gene expression which finally accounts for the different phenotypes of the two cell types. A cell-type specific gene expression does not necessarily result from a cell-type specific activation of these genes but can also come from a cell-type specific inhibition in the other cell-type. For example, it is known that the regulatory protein RegA acts on somatic cells to suppress gonidial development by inhibiting genes whose products are required for chloroplast biogenesis [9].

## Conclusion

The results show that quantitative real-time RT-PCR is a favorable approach to analyze cell-type specific gene expression in *Volvox carteri*. Our approach not only provides a basis for a detailed analysis of individual, previously unknown *Volvox *genes of the investigated set of genes but also allows for future analysis of the same set of genes (by using the same primers and other RT-PCR conditions) with respect to inducibility by stress, wounding, deficiency or abundance of nutritional compounds, or response on the presence of the sex-inducer (the trigger of sexual development in *V. carteri*). Furthermore, it allows a characterization of the transcription of these genes in the life cycle (probably without separating cell-types because a separation of cell types from embryos or juveniles can not be achieved earlier than 18–20 h after the onset of embryogenesis, and embryonic and parent somatic cells can't be separated from each other even later [6]. It is also possible to repeat these experiments using developmental or metabolic mutants instead. Finally, this approach can also be extended to a much larger number of genes. We hope that our analysis of cell-type specific expression of almost 40 genes was able to stimulate discussion about the application of a genome-wide expression analysis in *Volvox *in order to reveal the complete germ-soma program of this fascinating green alga.

## Methods

### Culture conditions

The wild-type *Volvox carteri *f. *nagariensis *strain EVE (female) was obtained from D.L. Kirk (Washington University, St. Louis, MO) and was described previously [46]. Cultures were grown in *Volvox *medium [47] at 30°C in an 8 h dark/16 h light (10000 lux) cycle [48].

### Sequence analysis and homology search

The sequences of cDNA and genomic DNA fragments were compared with each other to exclude duplicates and to identify overlapping sequences which belong to the same gene by using DNASIS software (version 7.00; Hitachi Software Engineering, South San Francisco, CA). Homology searches with cDNA and genomic DNA fragments in different sequence databases were performed using BLAST [49]. Initially, *Volvox carteri *f. *nagariensis *whole-genome shotgun reads at the *Chlamydomonas reinhardtii *web site (version 3.0) of the Joint Genome Institute (JGI) [5] were screened using BLASTn and blocks substitution matrix 62 (BLOSUM62) [50] for pairwise sequence alignment with a cut-off expectation value (E-value) of 10^-5 ^and a word size of 3 (filtering disabled). Subsequently, the sequence databases of the National Center for Biotechnology Information (NCBI) [51] were searched for homologous protein sequences using tBLASTx with an expect threshold of 10, a word size of 3, gap costs of 11 for opening a gap, and gap costs of 1 for extending a gap (filtering disabled). Finally, the *Chlamydomonas *EST database at the *Chlamydomonas reinhardtii *web site (version 3.0) of JGI [5] was screened using tBLASTx and the BLOSUM62 scoring matrix with a cut-off E-value of 10^-5 ^and a word size of 11 (filtering disabled).

### Primer design

Oligonucleotide primers for all PCR, standard RT-PCR or real-time RT-PCR were designed using the primer analysis software Oligo 6 (Molecular Biology Insights, Cascade, CO), Primer Express (Applied Biosystems, Foster City, CA), or DNASIS software (version 7.00; Hitachi Software Engineering, South San Francisco, CA). The primers used for real-time RT-PCR experiments are listed in Table 2.

### Large-scale separation of cell types

Shortly before the onset of cell cleavage of reproductive cells (gonidia), 10-liter cultures of synchronously grown *V. carteri *spheroids were harvested by filtration on a 100-μm mesh nylon screen, and the concentration of organisms was brought to ~1000 spheroids/ml with *Volvox *medium. To obtain gonidia, the spheroids were broken up in a 50 ml Dounce homogenizer with a tight-fitting pestle (B. Braun, Melsungen, Germany) by moving the pestle up and down twice. The cell suspension was filtered through a 100 μm nylon screen, and the flow-through was filtered through a 40 μm nylon screen. Only free gonidia, single somatic cells, and small cell sheets containing several ECM-embedded somatic cells can pass the 40 μm nylon screen, whereas larger cell sheets, hemispheres of the spheroids, or spheroids which have only been slit remain on the nylon screens. The gonidia were separated from most residual somatic cells and ECM fragments by centrifuging for 5 min at 350 g in 7% (v/v) Percoll (Sigma-Aldrich, St. Louis, MO). Individual somatic cells that remained after this procedure were removed from the gonidia by filtering through a 10 μm nylon screen. Single somatic cells pass this screen, in contrast to gonidia. The gonidia were washed on the screen three times with 100 ml *Volvox *medium each and were used for the gonidial RNA preparation.

To obtain somatic cells, spheroids were broken up in the 50 ml Dounce homogenizer with a tight-fitting pestle as described above, exept the pestle was moved up and down seven times. The obtained cell suspension was diluted with medium to the two-fold volume and kept at room temperature for 20 min. Gonidia and larger fragments of spheroids that contain gonidia settled during this time by unit gravity. By contrast, somatic cell sheets without gonidia floated to the surface, were drawn off, and were used for the somatic RNA preparation.

### Isolation of total RNA

Extraction of total RNA was done using 1 g frozen gonidia or somatic cells, 10 ml of the phenol-based TRI Reagent (Sigma-Aldrich, St. Louis, MO), and 3 ml trichloromethane. RNA was precipitated from the aqueous phase with isopropanol. RNA pellets were washed twice with 75% ethanol, air dried, and dissolved in RNase-free (DEPC treated) distilled water. RNA quantitation and purity check were done by agarose-formaldehyde gel electrophoresis and by measuring absorption at 260 and 280 nm with an Ultrospec 2100 pro UV/Visible Spectrophotometer (GE Healthcare, Uppsala, Sweden).

### Isolation of genomic DNA

Genomic DNA was isolated from *Volvox *algae using the DNeasy Plant Mini Kit (Qiagen, Hilden, Germany). DNA quantitation and purity check were done by agarose gel electrophoresis and UV-photometry using an Ultrospec 2100 pro UV/Visible Spectrophotometer (GE Healthcare, Uppsala, Sweden).

### Genomic PCR

Genomic PCR was carried out in a total volume of 25 μl containing ~100 ng of genomic DNA, 1 μM of each primer, 0.1 mM dNTP, and 2 units Taq DNA polymerase in 1× PCR reaction buffer (20 mM Tris-HCl pH 8.8, 10 mM KCl, 10 mM (NH_4_)_2_SO_4_, 2 mM MgSO_4_, 1 % Triton X-100, 1 mg/ml BSA). Forty cycles (95°C, 20 s; 55°C, 30 s; 72°C, 1 min) were performed with a T3 Thermocycler PCR system (Biometra, Göttingen, Germany). The lengths of PCR products were determined by comparison with DNA size markers (100 bp DNA marker, Fermentas, St. Leon-Rot, Germany and 1 kb DNA ladder, Invitrogen, Carlsbad, CA). Products of PCR amplification were cloned into the pBluescript II SK vector (Stratagene, La Jolla, CA) and sequenced.

### Standard RT-PCR

For standard RT-PCR, first strand cDNA synthesis was performed using Moloney murine leukemia virus (MMLV) reverse transcriptase lacking ribonuclease H activity (H minus) (Promega, Madison, WI). 1 μg total RNA was incubated with 10 pmol of a specific reverse primer in a total volume of 14 μl at 70°C for 5 min and cooled immediately on ice for 5 min. After addition of 5 μl MMLV RT 5× reaction buffer (250 mM Tris-HCl pH 8.3 at 25°C, 250 mM KCl, 20 mM MgCl_2_, 50 mM DTT), 1.25 μl 10 mM dNTPs, and 200 units MMLV RT (H minus), cDNA synthesis was performed at 50°C for 60 min in a total volume of 25 μl. PCR was subsequently carried out using 10 μl of the reverse transcription mixture, 1 μM of each primer, 0.2 mM dNTPs, and 2.5 units Taq DNA polymerase in 1× PCR reaction buffer in a total volume of 50 μl. PCR was performed on a T3 Thermocycler PCR system (Biometra, Göttingen, Germany) using the following cycling conditions: 45 cycles of 95°C for 20 s, 55°C for 30 s, and 72°C for 40 s. Products of RT-PCR amplification were cloned into pSPT18/pSPT19 vectors (Roche, Penzberg, Germany) and sequenced.

### Real-time RT-PCR

1 μg total RNA was treated with 5 units DNaseI (Promega, Madison, WI) in DNase-I buffer (20 mM Tris, pH 8.4, 2 mM MgCl_2_, 50 mM KCl) in a total volume of 10 μl at 37°C for 10 min to remove contaminating DNA within the RNA preparation. The reaction was stopped by the addition of 1 μl 25 mM EDTA and incubation at 65°C for 10 min. Real-time quantification of RNA targets was done using the QuantiTect SYBR Green RT-PCR Kit (Qiagen, Hilden, Germany). Use of 2× QuantiTect SYBR Green RT-PCR Master Mix together with the QuantiTect RT Mix allows both reverse transcription and PCR to take place in a single tube. The components of 2× QuantiTect SYBR Green RT-PCR Master Mix include HotStarTaq DNA Polymerase, QuantiTect SYBR Green RT-PCR buffer, the fluorescent dye SYBR Green I, and the passive reference dye ROX. The QuantiTect RT Mix contains Omniscript and Sensiscript Reverse Transcriptases. Reactions contained 300 ng DNase-I-treated template RNA, 0.8 μM of each primer, 12.5 μl 2× QuantiTect SYBR Green RT-PCR Master Mix, and 0.25 μl QuantiTect RT Mix in a total volume of 25 μl. Reverse transcription occured at 50°C for 30 min. The subsequent incubation at 95°C for 15 min denatured the cDNA template, deactivated the reverse transcriptases, and activated the HotStarTaq DNA Polymerase. After this, 40 cycles of PCR amplification (95°C, 20 sec; 55°C, 30 sec; 72°C, 40 sec) followed. All real-time RT-PCR reactions were performed using a DNA Engine Opticon Continuous Fluorescence Detection System (MJ research, Waltham, MA). This system excites fluorescent dyes with absorption spectra in the 450 to 495 nm range, like SYBR Green I, and sensitive optics detect fluorophores with emission spectra in the 515–545 nm range (like SYBR Green I). Results were analyzed using OpticonMonitor software (version 1.06, MJ research, Waltham, MA). All real-time RT-PCR experiments were carried out in triplicate together with RT minus (RTM) and no template controls (NTC). The final products of all real-time RT-PCR reactions were visualized by agarose gel electrophoresis to assure amplification of a single product and to verify the size of the cDNA products by comparison with a 100 bp ladder (100 bp Marker, Fermentas, St. Leon-Rot, Germany).

### Analysis of gene expression by using the 2^-ΔΔCt ^method

The expression level of a given target gene in gonidia versus somatic cells was analyzed using real-time RT-PCR and the 2^-ΔΔCt ^method [52,53]. The *Volvox *actin gene, which is known to be similarly expressed in both cell types [11], was used as an internal control in all real-time RT-PCR experiments. In order to apply the 2^-ΔΔCt ^method [52,53], the results of real-time RT-PCRs were represented as cycle threshold (C_t_) values. The C_t _value was defined as the cycle at which a sample crosses a threshold which is significantly above the background fluorescence and within the exponential phase of the amplification. The average from three C_t _measurements was calculated for both the given target gene and the actin gene. ΔC_t _was determined as the mean of the triplicate C_t _values for the target genes minus the mean of the triplicate C_t _values for the actin gene. For each target gene, ΔC_t _measurements were performed separately for each cell type. The ΔΔC_t _represented the difference between the two cell types for a given target gene, more precisely ΔΔC_t _= ΔC_t (gonidia) _- ΔC_t (somatic cells)_. The ×-fold higher expression of a given target gene in gonidia compared to somatic cells was calculated as 2^-ΔΔCt^. If expression of a given target gene was lower in gonidia as compared to somatic cells, the expression was calculated by 1/2^-ΔΔCt^.

### GenBank accession numbers

All 28 novel sequences described in this study have been deposited in GenBank under the following accession numbers:

*dyhA *[GenBank: EF123072], *klpA *[GenBank:EF123073],

*fer1 *[GenBank:EF123074], *nab1 *[GenBank:EF123075],

*rap41 *[GenBank:EF123076], *fbp1 *[GenBank: EF123077],

*cp12 *[GenBank: EF123078], *prfA *[GenBank: EF123079],

*fsd1 *[GenBank: EF123080], *rpl37 *[GenBank: EF123081],

*glu1 *[GenBank: EF123082], *hsp70B *[GenBank: EF123083],

*hsp40A *[GenBank: EF123084], *ubcA *[GenBank: EF123085],

*ponA *[GenBank: EF123086], *mat3 *[GenBank: EF123087],

*vpeA *[GenBank: EF123088], *sac1 *[GenBank: EF123089],

*rcd1 *[GenBank: EF123090], *adcA *[GenBank: EF123091],

*nipA *[GenBank: EF123092], *lciB *[GenBank: EF123093],

*upf1 *[GenBank: EF123094], *upf2 *[GenBank: EF123095],

*upf3 *[GenBank: EF123096], *upf4 *[GenBank: EF123097],

*upf5 *[GenBank: EF123098], and *upf6 *[GenBank: EF123099].

## Abbreviations

BLAST – basic local alignment search tool; cDNA – complementary DNA; C_t _– cycle threshold; EST – expressed sequence tag; gDNA – genomic DNA; PCR – polymerase chain reaction; RT-PCR – reverse transcription-polymerase chain reaction

## Authors' contributions

GN and AK were responsible for homology search, sequence analysis, primer design, and realization of all experiments. AH (corresponding author) conceived and coordinated the study, critically evaluated the data, did the final calculations, and wrote the manuscript. All authors read and approved the final manuscript.
